# Ornidazole suppresses CD133^+^ melanoma stem cells via inhibiting hedgehog signaling pathway and inducing multiple death pathways in a mouse model

**DOI:** 10.3325/cmj.2022.63.461

**Published:** 2022-10

**Authors:** Gulsah Evyapan, Umit Luleyap, Halil Mahir Kaplan, Ismail Oguz Kara

**Affiliations:** 1 Department of Medical Biology, Cukurova University Faculty of Medicine, Adana, Turkey; 2 Department of Pharmacology, Cukurova University Faculty of Medicine, Adana, Turkey; 3 Department of Medical Oncology, Cukurova University Faculty of Medicine, Adana, Turkey

## Abstract

**Aim:**

To evaluate the inhibitory effects of ornidazole on the proliferation and migration of metastatic melanoma cell line (B16F10) *in vitro* and its anti-cancer effects *in vivo* using a melanoma mouse model.

**Methods:**

We investigated the effects of ornidazole on cell viability (Crystal Violet and MTT assay) and migration ability (wound-healing assay) of B16F10 melanoma cells, and its ability to trigger DNA damage (Comet assay) *in vitro*. We also sorted CD133^+^ and CD133^-^ cells from B16F10 melanoma cell line and injected them subcutaneously into Swiss albino mice to induce tumor formation. Tumor-bearing mice were divided into control and treatment groups. Treatment group received intraperitoneal ornidazole injections. Tumors were resected. Real-time polymerase chain reaction was used to determine the expression of genes involved into Sonic hedgehog (Shh) signaling pathway, stemness, apoptosis, endoplasmic reticulum (ER) stress, ER stress-mediated apoptosis, and autophagy. Shh signaling pathway-related proteins and CD133 protein were analyzed by ELISA.

**Results:**

Ornidazole effectively induced DNA damage in CD133^+^ melanoma cells and reduced their viability and migration ability *in vitro*. Moreover, it significantly suppressed tumor growth in melanoma mouse model seemingly by inhibiting the Shh signaling pathway and ER-stress mediated autophagy, as well as by activating multiple apoptosis pathways.

**Conclusions:**

Our preclinical findings suggest the therapeutic potential of ornidazole in the treatment of metastatic melanoma. However, larger and more comprehensive studies are required to validate our results and to further explore the safety and clinical effectiveness of ornidazole.

Melanoma is a highly metastatic skin cancer developing as a result of malignant transformation of melanocytes and one of the fastest growing malignancies worldwide (1-4). Although the five-year survival rate of melanoma is considerably high (>95%) when diagnosed early, melanoma prognosis is extremely poor once the disease becomes metastatic (5,6). Conventional chemotherapies, generally aimed at inhibiting cell division, have shown little survival benefit. The most important cause of treatment failure is resistance to conventional chemotherapy and radiotherapy (7-9). Chemotherapy resistance in advanced-stage melanoma is associated with a subset of CD133^+^ melanoma-initiating cells (10), a type of cancer stem cells (CSCs) (11-13). Thus, eradication of CSCs is an important goal in therapeutic approaches because it could dramatically reduce the risk of metastatic dissemination and relapses, the major causes of mortality in oncology patients (14).

Among CSC markers, CD133 (Prominin-1), a pentaspan membrane glycoprotein, has been considered as one of the most important surface markers for identification of melanoma stem cells (15,16). High levels of CD133 expression have been linked to the high tumorigenicity and metastatic potential of melanoma cells (17-19). Moreover, CD133 promotes metastasis via interaction with signaling pathways that regulate cell migration and polarity dynamics (17,20). Indeed, the crosstalk between several different signaling pathways, including Sonic hedgehog (Shh) (21,22), plays a role in the pathogenesis and progression of malignant melanoma (23,24). Therefore, anti-cancer treatments targeting the Shh pathway show promise in many human cancers, including melanoma, lung, breast, and prostate cancers (25-27).

Over the last decade, novel treatment strategies, including immunotherapy and targeted therapy, targeting the key regulators of these signaling pathways (28-31) have resulted in some improvement in the setting of metastatic melanoma (32). Despite this, patients with melanoma often experience disease progression and relapse with acquired resistance after several months of monotherapy, and the five-year survival rate for the metastatic disease remains low (6,33). Development of new effective and selective therapeutic compounds that specifically target melanoma-specific CSCs and related signaling pathways is therefore needed (14).

Ornidazole has been used for the treatment of infections caused by anaerobic bacteria and protozoa in humans through a mechanism that includes pre-activation by the reduction of the nitro groups and the production of toxic derivatives and radicals (34). Ornidazole enters the cell by passive diffusion under anaerobic conditions and inhibits DNA synthesis by breaking and destabilizing the DNA structure (35). The effects of ornidazole on melanoma cells and the mechanisms through which it may influence the tumor structure in melanoma have not been investigated. Given that tumor cells also create anaerobic environments, we hypothesized that ornidazole may be a promising compound for the treatment of malignant melanoma. To test this hypothesis, we evaluated the inhibitory effects of ornidazole on the proliferation and migration of metastatic melanoma cell line (B16F10) *in vitro* and its anti-cancer effects *in vivo* using a melanoma mouse model.

## MATERIAL AND METHODS

### Cell lines and reagents

B16F10 mouse melanoma cancer cell line was purchased from ATCC (CRL-6475, Manassas, VA, USA). Ornidazole was obtained from Sigma (cat. no: 16773-42-5, Sigma, Roedermark, Germany) and was used after having been dissolved in Dulbecco's Modified Eagle Medium (DMEM) and distilled water at room temperature for *in vitro* and *in vivo* assays, respectively.

### Cell culture

Cells were maintained in DMEM (cat. no: 11965084, ThermoFisher, Waltham, MA, USA) supplemented with 100 U/mL penicillin (cat. no: P4443, Sigma), 100 mg/mL streptomycin (cat. no: 85886, Sigma), and 10% fetal bovine serum (FBS) (cat. no: P2442, Sigma) in a humidified incubator at 37 °C under 5% CO_2_ atmosphere.

### Crystal Violet assay for determining the viability of melanoma cells

Crystal Violet staining was performed to observe the effects of ornidazole on the viability of B16F10 cells. Cells were seeded in a 12-well plate at a density of 70 000 cells/well and were treated with ornidazole at various concentrations (400, 800, and 1200 μg/mL) under humidified atmosphere containing 5% CO_2_ at 37 °C for 48 h. The treated cells were then stained with 0.5% Crystal Violet staining solution (cat. no: 548-62-9, Sigma) at room temperature for 30 minutes. After the plate was gently rinsed with water and dried, the absorbance values of each well were determined spectrophotometrically at 570 nm by using a plate reader (Biochrom, Cambourne, UK).

### Cell growth inhibition by ornidazole

The effects of ornidazole on tumor cell growth were assessed with the 3-(4,5-dimethylthiazol-2-yl)-2,5-diphenyltetrazolium bromide (MTT) assay (cat. no: CT01-5, Sigma). The MTT assay was performed as per the manufacturer's protocol. Shortly, the cells were seeded into a 96-well plate at a density of 10 000 cells/well and cultured for 24 h, 48 h, and 72 h, in the presence or absence of ornidazole at various concentrations (50, 100, 200, 800, 1200, 1600, 3600 μg/mL). The cells that were not treated with ornidazole were used as a control group, and an equal amount of DMEM medium was added to the control well plates. Following incubation at 37 °C for 4 h, 100 μL of solubilizing buffer was added to each well. After overnight incubation, the absorbance values of each well were determined with an ELISA plate reader at 590 nm to assess cell viability.

In order to find the most effective dose of ornidazole (from the lowest dose to the lethal dose), more than one dose was selected at regular intervals taking into account the doses that act on various parasites. In addition, different drug concentrations from 0 to lethal dose (maximum concentration) were tested with increasing drug doses in the MTT assay, and the most suitable concentrations were selected for treatment.

### Wound-healing assay

The effect of ornidazole on cell migration was evaluated with wound-healing assay (36). In short, B16F10 cells were seeded into six-well plates with a density of 80 000 cells/well and allowed to grow to 80%-90% confluence. Next, the cells were washed with phosphate buffered saline (PBS) and cultured in DMEM containing 0.05% fetal bovine serum (FBS) for 16 h. A scratch/wound with clear edges was created across the width of the well with a 200-μL pipette tip and was washed twice with PBS to remove debris or detached cells. The cells then were exposed to different concentrations of ornidazole (0, 400, 800, 1200 μg/mL). Cell migration was photographed at 0, 8, and 24 h with a digital camera installed on the microscope (Leica, Wetzlar, Germany). The wound area was measured with the Image J software (NIH, Bethesda, MD, USA).

### Comet assay for determining DNA damage

Ornidazole has been reported to efficiently induce DNA damage under the anaerobic conditions (37). Therefore, we reasoned that the hypoxic tumor microenvironment might facilitate the access of ornidazole into the tumor cells to create DNA damage. The Comet assay was performed to evaluate the double-strand breaks (DSBs) in the DNA of B16F10 melanoma cells.

The cancer cells were treated with ornidazole at various concentrations (0, 400, 800, and 1200 μg/mL) for 24 h, 48 h, and 72 h. Treated cells were then mixed with low-melting-point agarose, and the mixture was spread on the frosted microscope slides precoated with a thin layer of normal-melting-point agarose. The slides were exposed to lysis buffer (2.5 M NaCl, 1% Triton X-100, 100 mM EDTA, 10 mM Tris-HCI, pH 10) at 4 °C for 1 h. Then, the electrophoresis chamber was filled with cold electrophoresis buffer (300 mM NaOH, 1 mM EDTA, pH: 13), and the slides were kept in the buffer for 40 min at 4 °C to allow DNA unwinding. After DNA unwinding, the slides were subjected to electrophoresis for 20 min at 25 V. Then, the slides were washed with neutralization buffer (0.4 M Tris-HCI, pH: 7.5) for 15 min and with 96% ethanol for 10 min. All preparatory steps were carried out in the dark to avoid additional DNA damage (38).

After ethidium bromide staining, the slides were viewed at 40 × magnification, and the Comet tails were visualized with a UV microscope (Leica). The degree of DNA damage was assessed based on the tail length and distribution of DNA in the tail, which is known as the olive tail moment (OTM). The measurements were derived from 100 tumor cells per sample group. The data were analyzed with Comet Score software (CaspLab, Wroclaw, Poland).

### Sorting of CD133^+^ cancer stem cells with flow cytometry

CD133^+^ cells on B16F10 cell line were identified and isolated as previously described (39). In short, cells were detached using PBS, resuspended in PBS, and incubated with PE-conjugated mouse anti-human CD133 antibody (clone: 315-2C11, Biolegend, San Diego, CA, USA) at 1:100 dilution at room temperature for 30 minutes in the dark. Samples were acquired on FACSAria III flow cytometer (Becton Dickinson, Beckman Coulter, Inc., Brea, CA, United States), and CD133^+^ or CD133^-^ cells were sorted into DMEM medium.

### Serum-free culture for cancer stem cells

After sorting, cells were centrifuged, rinsed, and seeded in 6-well plates into serum-free medium supplemented with 20 μg/L basic fibroblast growth factor-basic (bFGF), 10 μg/L epidermal growth factor (EGF), penicillin G (100 U/mL), and streptomycin (100 μg/mL). The culture medium was changed every two days, and cell proliferation was observed under an inverted phase-contrast microscope, at 0 h, 24 h, 48 h, and 72 h.

### Animal model

Thirty-six male Swiss albino mice, 8 weeks of age, were purchased from the Cukurova University Health Sciences Experimental Application and Research Center and divided into three groups each consisting of 12 mice: 1) unsorted group (6 treatment and 6 control mice), 2) CD133^+^ group (6 treatment and 6 control mice), and 3) CD133^-^ group (6 treatment and 6 control mice). All mice were kept under the following conditions: temperature 21-23 °C, humidity 70%, and 12 h light-dark cycle with food and water *ad libitum*. To minimize animal suffering, all mice were anesthetized with isoflurane (2%-2.5%) before euthanasia.

### Melanoma cells injection

Cells were incubated in a humidified 37 °C incubator with 5% CO_2_. For melanoma cancer model, unsorted B16F10 cells (5x10^5^), CD133^+^ cells (1x10^5^), and CD133^-^ cells (5x10^5^) were resuspended in 100 μL of DMEM and subcutaneously injected into the Swiss albino mice (n = 36). Tumor length and width were measured three times a week with Vernier Calipers (40). The measurements were made between the longest longitudinal (length) and the longest transverse (width) sections. The long section was considered as the tumor length and the short section was considered as the tumor width. The tumor volume was calculated by the following formula: (width × width × length)/2 (40). After tumor volume reached ~ 100-150 mm^3^, intraperitoneal (IP) treatment with ornidazole (80 mg/kg body weight) was delivered to six mice (treatment arm) in each group daily for 12 days. The remaining six mice in each group did not receive ornidazole treatment.

### Real-time polymerase chain reaction analysis

We used RT-PCR to analyze whether ornidazole treatment affected the expression levels of hedgehog signaling pathway-related genes, including *Shh*, *Smo, Gli1, Ptch1,* and *Bmi1* in tumor tissues.

Next, we examined whether ornidazole altered the expression of significant CSC-related genes, including *Prom1* (gene encoding CD133), *Oct3/4, Nanog*, and *Sox2*, in unsorted, CD133^+^ and CD133^-^ tumor cells.

As the BCL2 family plays a critical role in the execution of programmed cell death, we determined whether ornidazole-induced apoptosis was associated with changes in *Bax, Casp3, Casp9* and *Bcl2*.

We also explored whether ornidazole activated other apoptosis-related pathways in melanoma tumors, including *Grp78* and *Xbp1* as well as ER stress-mediated apoptosis markers, such as *Chop* and *Casp12*. As ER stress is also a potent inducer of autophagy process in the cells (41), we examined the expressions of autophagy-related genes, including *Atg5, Atg12, Becn1*, *Map1lc3b*, and *Atf4* in unsorted, CD133^+^, and CD133^-^ groups.

RNA was prepared by using TRIzol (Invitrogen, Waltham, MA, USA). cDNA was prepared from 2 μg of total RNA in a 20-μL RT reaction by using the Applied Biosystems High-Capacity cDNA kit (ThermoFisher Inc., Waltham, MA, USA). *β-actin* was selected as the reference gene. The primers used are listed in Table 1.

**Table 1:** Sequences of the mouse primers used in real-time polymerase chain reaction

Gene	Forward primer	Reverse primer
*Gli1*	CCAAGCCAACTTTATGTCAGGG	AGCCCGCTTCTTTGTTAATTTGA
*Shh*	AAAGCTGACCCCTTTAGCCTA	TGAGTTCCTTAAATCGTTCGGAG
*Smo*	CTTGGTGCGAACAGACAACC	GGTAGCGATTGGAGTTCCGC
*Ptch1*	TGCCACAGCCCCTAACAAAAA	ACCCACAATCAACTCCTCCTG
*CD133*	ACTGGGGCTGTGTGGAAAG	GCATTGAAGGTATCTTGGGTCTC
*Sox2*	CAATCCCATCCAAATTAACGCA	AAGCTGCAGAATCAAAACCC
*Bmi1*	AAATCCCCACTTAATGTGTGTCC	CTTGCTGGTCTCCAAGTAACG
*OCT3/4*	AGAGGATCACCTTGGGGTACA	CGAAGCGACAGATGGTGGTC
*Nanog*	TCGCCCTTCCTCTGAAGAC	TGCTTCTGAAACCTGTCCTTGA
*Xbp-1*	GACAGAGAGTCAAACTAACGTGG	GTCCAGCAGGCAAGAAGGT
*GRP78*	GTGTTCAAGAACGGCCGCGTG	GTTTGCCCACCTCCAATATCAAC
*ATF4*	CTCTTGACCACGTTGGATGAC	CAACTTCACTGCCTAGCTCTAAA
*CHOP (Ddit3)*	CTCGCTCTCCAGATTCCAGTC	CTTCATGCGTTGCTTCCCA
*ATG5*	AGCCAGGTGATGATTCACGG	GGCTGGGGGACAATGCTAA
*Becn1*	ATGGAGGGGTCTAAGGCGTC	TGGGCTGTGGTAAGTAATGGA
*Map1LC3b*	CGGAGCTTTGAACAAAGAGTG	TCTCTCACTCTCGTACACTTC
*ATG12*	TGCTGAAGGCTGTAGGAGACACTC	TGATGAAGTCAATGAGTCCTTGGATGG
*Casp12*	ATGCTGGATTGGCCCATGAAT	AGACGTGTTCGTCCCTCCTT
*Bcl2*	GCTACCGTCGTGACTTCGC	CCCCACCGAACTCAAAGAAGG
*Bax*	AGACAGGGGCCTTTTTGCTAC	AATTCGCCGGAGACACTCG
*Casp3*	CTCGCTCTGGTACGGATGTG	TCCCATAAATGACCCCTTCATCA
*Casp9*	GGCTGTTAAACCCCTAGACCA	TGACGGGTCCAGCTTCACTA
*Actb*	GTGTGACGTTGACATCCGTA	GTACTCCTGCTTGCTGATCC

Polymerase chain reactions (PCR) were performed with Power SYBR Green PCR Master Mix (ThermoFisher Inc.) on a 96-well reaction plate, and Applied Biosystems StepOnePlus Real-Time PCR System (Applied Biosystems, Waltham, MA, USA) was used for quantification of gene expression. The quantitative PCR conditions were as follows: 50 °C for 2 min, 95 °C for 10 min, 40 cycles of 95 °C for 30 seconds, and 60 °C for 1 min). Each biological sample was run in triplicate on the same reaction plate, and the average Ct values obtained from technical replicates were used for final calculation.

### Enzyme-linked immunosorbent assay (ELISA)

Hedgehog signaling pathway-related proteins and CD133 protein were analyzed with ELISA kit (SunredBio Inc., Shanghai, China) according to the manufacturer's instructions. For total protein extraction, tumor tissues were treated with radioimmunoprecipitation assay and 0.3% (v/v) protease inhibitor (cat no: 8778, Sigma-Aldrich) and homogenized with an electric homogenizer on ice. Next, the lysates were centrifuged at 10 000 rpm for 10 min at 4 °C. The relative concentration of each protein in the supernatant was determined with appropriate ELISA kits.

### Statistical analysis

The normality of distribution for continuous variables was confirmed with the Shapiro Wilk test. Differences between the groups in Crystal Violet and MTT assays results were assessed with a one-way ANOVA with multiple comparisons and a post-hoc Dunnet's test. Differences in wound-healing assay results were assessed with a one-way ANOVA with Bonferroni correction. Differences in Comet assay results, as well in tumor volumes in experimental melanoma models, were assessed with a two-way ANOVA with Bonferroni correction. The differences in gene expression were assessed with one-way ANOVA with Bonferroni correction. For data obtained by ELISA assay, two-way ANOVA with Bonferroni correction was performed. Different doses of ornidazole treatment were compared with the control group, and the data are presented as mean ± standard deviation. The level of significance was set at 0.05 (*P* < 0.05). Data analysis was performed with IBM SPSS, version 20.0 (IMB Corp., Armonk, NY, USA) and Graphpad Prism, version 5.0. (GraphPad Software Inc, San Diego, CA, USA)

## RESULTS

### Ornidazole inhibited B16F10 melanoma cancer cells viability in a dose-dependent and time-dependent manner

**Figure 1:**
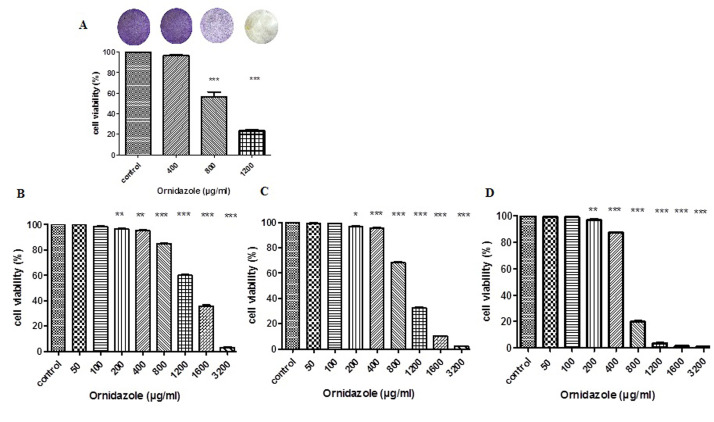
Results of Crystal Violet and MTT assays. **(A)** Cellular viability was assessed with Crystal Violet staining. Melanoma cancer cells were treated with the denoted concentrations of ornidazole for 48 h. **(B-D)** Ornidazole inhibits B16F10 melanoma cancer cells viability as determined by MTT assay. Melanoma cancer cells were treated with different concentrations of ornidazole for **(B)** 24 h, **(C)** 48 h, **(D)** 72 h. Results were presented as mean ± standard deviation of three independent experiments. Each group with different concentration of ornidazole was compared with the control group (one-way ANOVA with Dunnet's test for multiple comparisons, *P < 0.05; **P < 0.01; ***P < 0.001).

The MTT and Crystal Violet assays confirmed the inhibitory effects of ornidazole on the viability and proliferation of B16F10 melanoma cancer cells as the survival percentage of cells treated with ornidazole was lower compared with that of control cells. Crystal Violet assay showed that melanoma cells treated with ornidazole could not survive in the medium for 48 h, as opposed to control cells (Figure 1A, *P* < 0.001). MTT assay showed that ornidazole inhibited B16F10 cell proliferation in a concentration-dependent and time-dependent manner, with concentrations >200 μg/mL markedly reducing cell viability (Figure 1B, 1C, and 1D, *P* < 0.001 for all). An increase in the concentration of ornidazole from 200 μg/mL to 3200 μg/mL raised the inhibition rate from 5% to 97% during 72-h treatment (Figure 1D).

### Ornidazole suppressed the migratory and invasive abilities of melanoma cells

**Figure 2:**
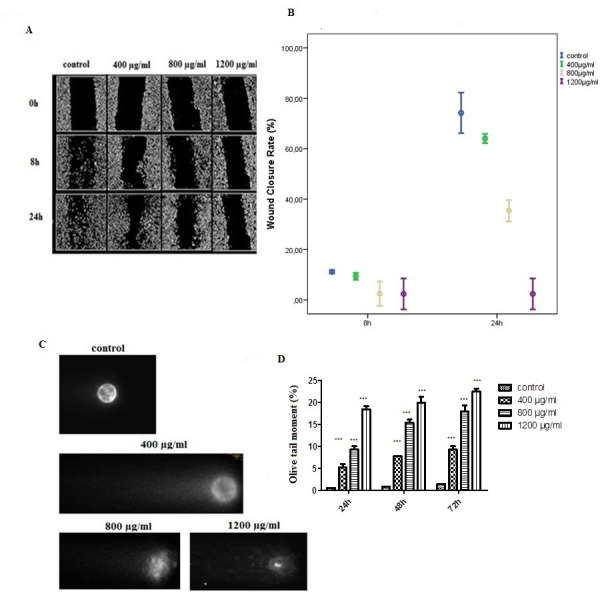
Wound-healing and Comet assays. The results are expressed as percentage of wound closure, and the area measured at time zero was considered 0% **(A)** Ornidazole inhibits B16F10 melanoma cancer cells migration as determined by wound-healing analysis. **(B)** Wound closure rates (%) at 8 h and 24 h. Ornidazole (400 μg/mL, 800 μg/mL, 1200 μg/mL) inhibited cell migration by 15%, 60%, and 96%, respectively, in B16F10 cells after 24 hours (one-way ANOVA followed by Bonferroni testing for multiple comparisons. Data are shown as the mean ± standard deviation, *P* < 0.05) **(C)** DNA damage as measured by the Comet assay as a result of treatment with different concentrations of ornidazole (400 μg/mL, 800 μg/mL, 1200 μg/mL) for 24, 48, and 72 hours. Olive moment = (tail mean-head mean) x % of DNA in the tail **(D)** Mean tail moment (μm) represents the damage distribution in the attached cells. The experiment was done in triplicates, and data are expressed as mean ± standard deviation. Each group was compared with the control group (two-way ANOVA with multiple comparisons, Bonferroni test was used for post hoc analysis, *** *P* < 0.001).

Ornidazole dose-dependently and significantly inhibited the migration of B16F10 cells compared with the untreated control (Figure 2A). Treatment with 400, 800, and 1200 μg/mL ornidazole inhibited B16F10 cells migration by 15%, 60%, and 96%, respectively, after 24 h (Figure 2A and 2B, *P* < 0.05). These results may also suggest that ornidazole inhibits the invasion potential of melanoma cells.

### Ornidazole induced DNA double-strand breaks in a dose-dependent and time-dependent manner

The Comet assay showed that ornidazole increased DNA damage in B16F10 melanoma cancer cells in a time-dependent and dose-dependent manner (Figure 2C and 2D) compared with the control group. Moreover, increasing the concentration of ornidazole from 400 μg/mL to 1200 μg/mL increased the olive tail moment (OTM) rate from 5% to 17% and from 9% to 22.5% during 24-h and 72-h treatment, respectively (*P* < 0.001).

### Ornidazole decreased tumor volume in unsorted, CD133^+^, and CD133^-^ cells

**Figure 3:**
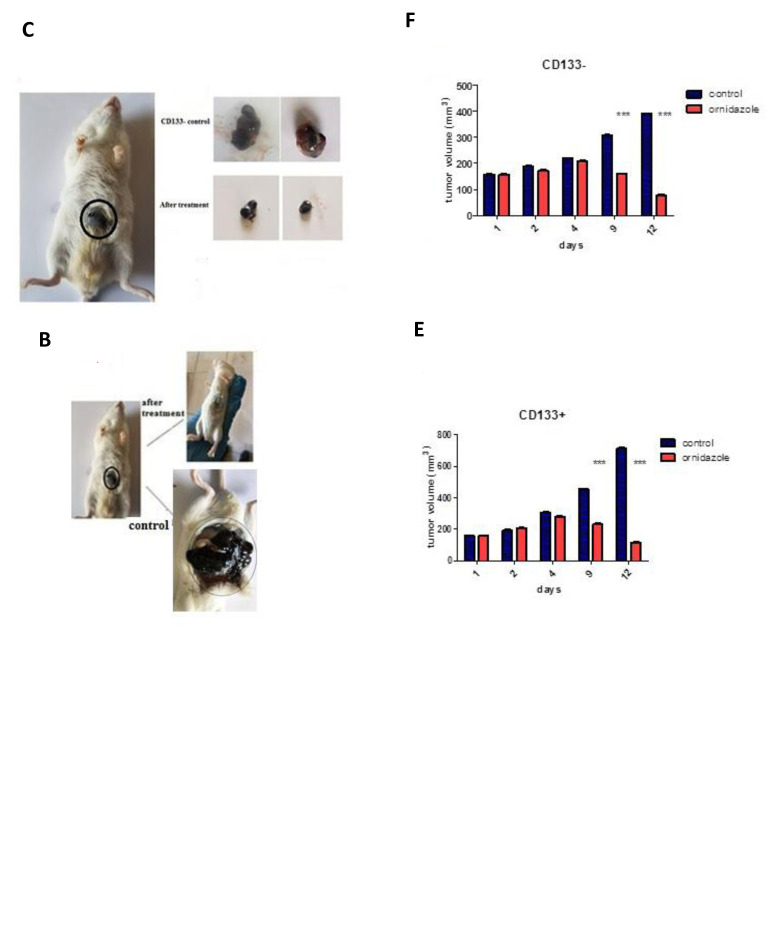
**(A)** Mice that were injected unsorted B16F10 cells and tumors resected from ornidazole-treated and control mice that received unsorted cell injection. **(B)** Mice that were injected CD133^+^ B16F10 cells. **(C)** Mice that were injected CD133^-^ B16F10 cells and tumors resected from ornidazole-treated and control mice that received CD133^-^ cell injection. **(D)** After ornidazole treatment, average tumor volume measurements of the unsorted group (***P < 0.001), **(E)** CD133^+^ (4th day **P < 0.01, 9th-12th day ***P < 0.001), and **(F)** CD133^-^ groups (2nd day *P < 0.5, 9th-12th day ***P < 0.001). All groups were compared with the control group (two-way ANOVA with Bonferroni correction for multiple comparisons).

All mice developed tumors in the injection site. However, the rate of tumor growth and the tumor volume were significantly higher in the CD133^+^-injected mice than in the CD133^-^ and unsorted groups (Figure 3F, 3H, and 3K). Furthermore, CD133^+^ cell-derived tumors created abnormally big metastasis around the injection site, whereas no metastasis was found in the mice who received CD133^-^ or unsorted cell injections (Figure 3D, 3G, and 3I). Therefore, our results confirmed a greater *in vivo* tumorigenic potential of CD133^+^ cells compared with that of CD133^-^ and unsorted B16F10 cells.

Mean tumor volumes significantly decreased in all treatment groups (unsorted, CD133^+^, and CD133^-^) compared with the control mice in the same group. In the unsorted group, three mice from the treatment arm completely recovered (complete loss of tumor tissue) and the other three had a 96% reduction in tumor tissue, while six control mice had a 200% increase in tumor volume (Figure 3D, 3E and 3F). In the CD133^+^ group, six treated mice showed 50% reduction in tumor volume, while 6 control mice had an increase in the tumor volume by 337.5% (Figure 3G and 3H). In the CD133^-^ group, in 6 treated mice tumor volume decreased, while in 6 control mice it significantly increased (150%) (Figure 3I, 3J, and 3K).

### Ornidazole treatment downregulated the genes associated with the hedgehog signaling pathway

**Figure 4:**
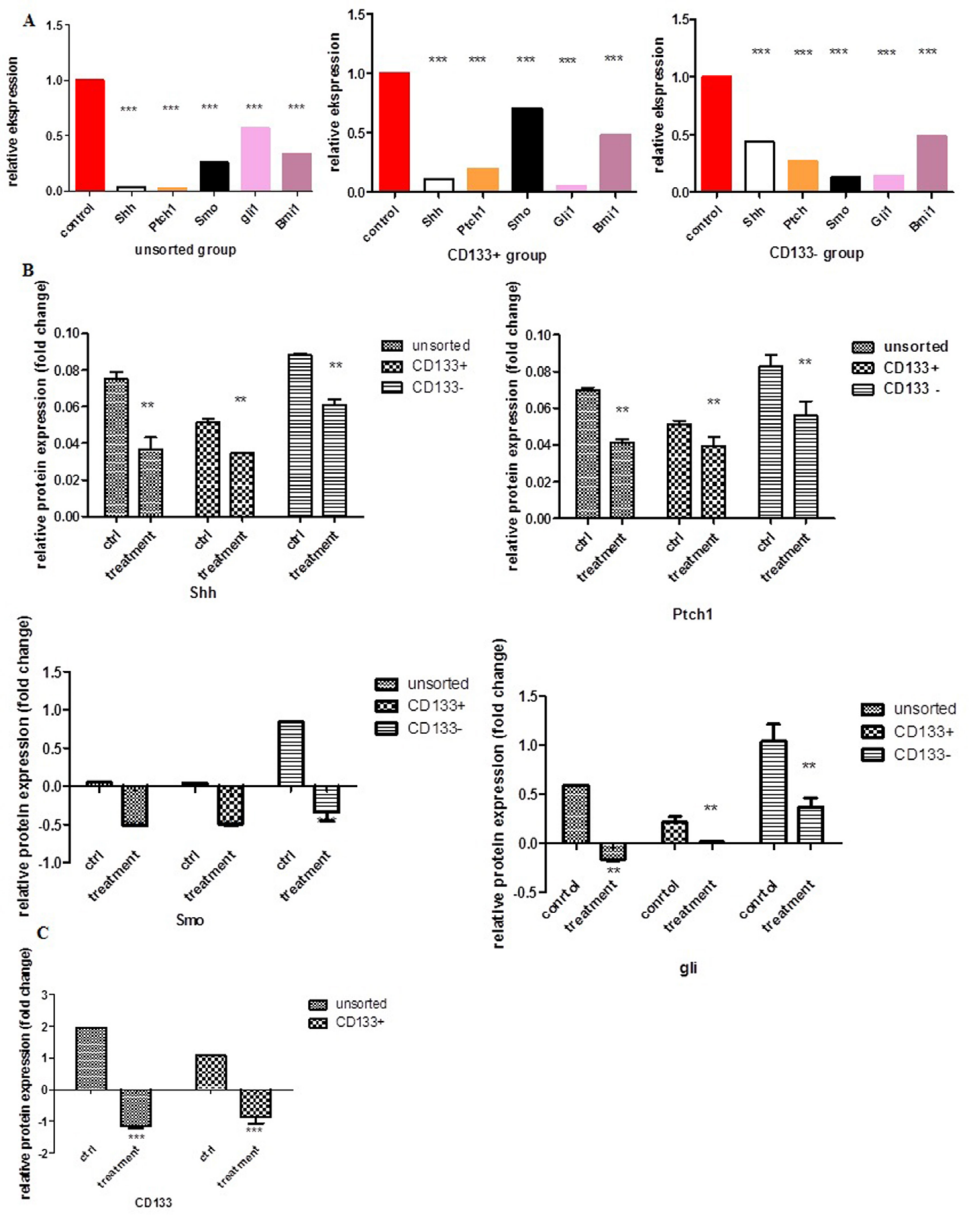
Gene and relative protein expression levels of hedgehog signaling pathway. **(A)** Effect of ornidazole on hedgehog signaling pathway in unsorted, CD133^+^, and CD133^-^ cells was assessed with real time-polymerase chain reaction. Tumor cells were isolated from mice. Each group was compared with the control group. Data are shown as mean ± standard deviation from three independent experiments (one-way ANOVA with Bonferroni corrections for multiple comparisons, *** *P* < 0.001, ** *P* < 0.01 (n = 6/group). **(B)** The fold-change levels of protein expression in ELISA assay of the B16F10 cells treated with ornidazole. The experiments were performed in duplicate. Each group was compared with the control group. Results are presented as mean ± standard deviation of three independent experiments (two-way ANOVA with Bonferroni testing for multiple comparisons, *** *P* < 0.001, **P < 0.01, n = 6 per group). Shh – Sonic hedgehog, PTCH1 – patched-1, SMO – smoothened, GLI1 – glioma-associated oncogene homologue-1, CD133 – also known as AC133 and prominin-1.

**Figure 5:**
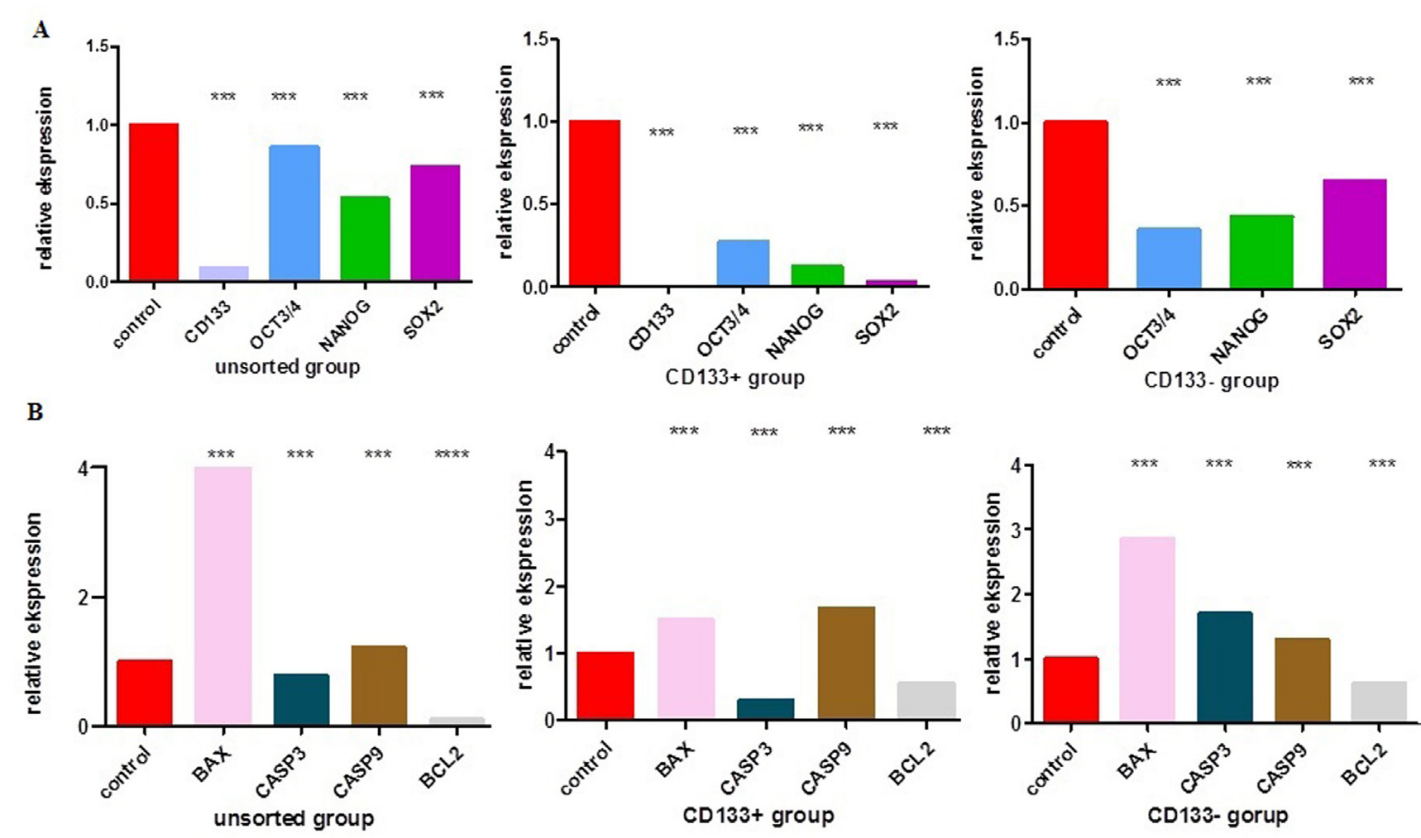
Gene expression of cancer stem cells- and apoptosis-related genes. **(A)** Effect of ornidazole on cancer stem cells marker in unsorted, CD133^+^, and CD133^-^ group was assessed with real time-polymerase chain reaction (RT-PCR). **(B)** The expression levels of apoptosis genes were analyzed with RT-PCR in unsorted, CD133^+^ and CD133^-^ groups. All groups were compared with control group. Results are presented as mean ± standard deviation of three independent experiments (one-way ANOVA followed by Bonferroni correction for multiple comparisons, *** *P* < 0.001, n = 6/group).

The expression levels of *Shh*, *Smo, Gli1, Ptch1,* and *Bmi1* (Figure 4A, *P* < 0.001), as well as those of SHH, PTCH1, SMO, GLI1 (Figure 4b) and CD133 (Figure 4c) proteins were significantly reduced in the treatment group compared with the control group. Therefore, both RT-PCR (Figure 4A and Figure 5A) and ELISA showed that ornidazole selectively downregulated CD133 and hedgehog signaling pathway-related genes in melanoma tumors.

### Ornidazole inhibited CD133, Nanog, Oct3/4, and Sox2 gene expression in tumor tissues

The expression levels of *CD133* (*P* < 0.001), *Oct3/4* (*P* < 0.001), *Nanog* (*P* < 0.001), and *Sox2* (*P* < 0.001) significantly decreased in the ornidazole-treated mice in all three groups compared with the control mice (Figure 5A).

### Ornidazole induces cell death in melanoma cells through GLI1/BCL2/BAx-axis as well as through ER-stress apoptosis

In all groups, ornidazole significantly increased *Bax* activation and decreased *Bcl2* activation (Figure 5B *P* < 0.001). Considering that ornidazole also significantly decreased both mRNA and protein expression level of *Gli1* (Figure 4A and 4B, *P* < 0.001), we believe that apoptosis induced by ornidazole in the melanoma tumor tissue might be partly mediated by Gli1/Bcl2/Bax-axis.

**Figure 6:**
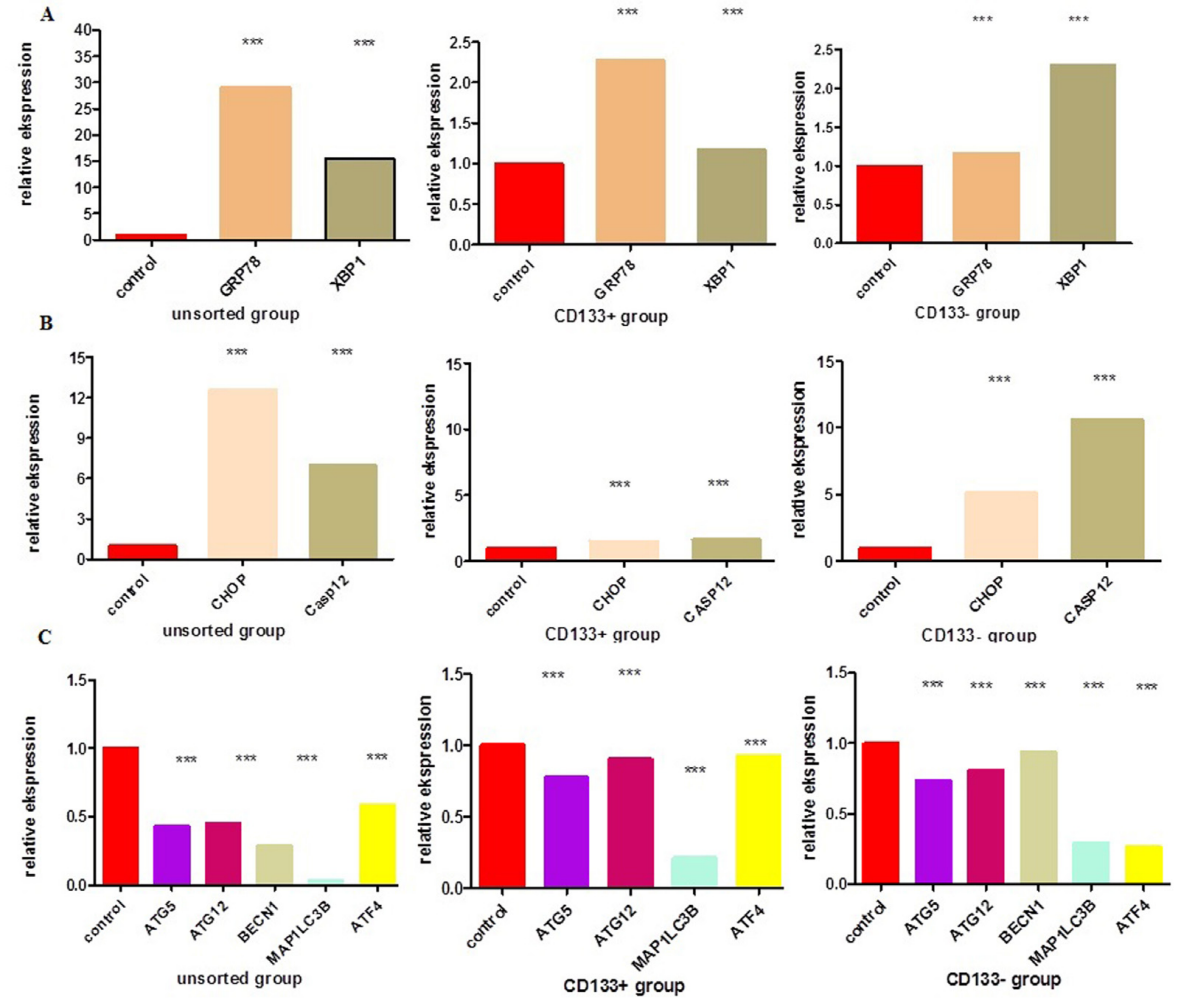
Expression levels of endoplasmic reticulum (ER) stress-associated, ER stress-mediated apoptosis, and autophagy-related genes. The expression levels of **(A)** ER stress related genes, **(B)** ER stress-mediated apoptosis genes, and **(C)** ER stress-mediated autophagy genes were analyzed with real time-polymerase chain reaction (RT-PCR) in unsorted, CD133^+^, and CD133^-^ groups. Each group was compared with the control group. Data are representative of three independent experiments, and the values are expressed as mean ± standard deviation (one-way ANOVA followed by Bonferroni correction for multiple comparisons, *** *P* < 0.001, n = 6/group).

Ornidazole increased the expression levels of cellular stress markers, including *Grp78* and *Xbp1* (Figure 6A, *P* < 0.001) as well as ER stress-mediated apoptosis markers, such as *Chop* and *Casp12*, in unsorted, CD133^+^, and CD133^-^ groups (Figure 6B, *P* < 0.001). Autophagy-related genes, including *Atg5, Atg12, Becn1*, *Map1lc3b*, and *Atf4,* in unsorted, CD133^+^, and CD133^-^ groups were significantly downregulated in ornidazole-treated cells compared with the control groups (Figure 6C, *P* < 0.001). Taken together, our results demonstrated that ornidazole might exert its anti-cancer effect in melanoma by inhibiting the autophagy machinery and by activating multiple apoptosis-related pathways.

## DISCUSSION

To our knowledge, this is the first study to explore the therapeutic potential of ornidazole for the treatment of malignant melanoma. Our *in vitro* experiments demonstrated that ornidazole efficiently and significantly inhibited cell viability and proliferation, suppressed migration capacity, and induced DNA damage in B16F10 melanoma cells. Furthermore, *in vivo* data showed that ornidazole treatment dramatically reduced tumor volume, specifically targeted CD133^+^ CSCs, upregulated pathways associated with cellular and ER-related stress, inhibited the Shh signaling and ER-stress mediated autophagy process, and activated two different apoptosis pathways in melanoma tumors.

Targeting the Shh signaling has been shown as a potential therapeutic approach for the treatment of some cancers, including melanoma (24,26,27,42). Interestingly, our study showed that the reduction in *Smo* and *Bmi1* expressions in CD133^+^ melanoma cell-injected mice, although significantly decreased compared with the control mice, was lower compared with other Shh-related genes. In a recent study, *Bmi1* induced an invasive signature that promoted metastasis and chemoresistance in melanoma (43). *Smo* has been shown to be involved in the pathogenesis and progression of many solid tumors, including breast, liver, pancreatic, and colon cancer. The overexpression of *Smo* has been linked to the tumor size, metastasis, invasiveness, and recurrence of disease, and SMO inhibitors have been used to suppress cancer formation, trigger apoptosis, and suppress cancer stem cell activity (44). Therefore, the downregulation of *Smo* and *Bmi1,* strong mediators of metastasis and recurrence, by ornidazole treatment highlights the therapeutic efficacy of ornidazole in melanoma setting.

The overexpression of the *Sox2*, *Oct4,* and *Nanog* genes is a general hallmark of a variety of human malignancies. These genes are associated with tumor invasion/metastasis, tumor formation, drug resistance, and disease recurrence after chemo/radiotherapy (10,45). In fact, *Sox2* contributes to melanoma cell invasion (46) and is a critical factor for self-renewal and tumorigenicity of melanoma-initiating cells (47). Targeting the molecular pathways in CSCs and downregulation of *CD133*, *Nanog*, *Oct3/4*, and *Sox2* have been crucial steps to control the tumor progression (19,37,48). We observed a significant decrease in the expression levels of these genes in the treatment groups compared with controls. This suggests that ornidazole can effectively target the CSC population in melanoma tumors.

In the last decades, developing treatment strategies that efficiently eliminate cancer cells and CSCs by apoptosis has become one of the major goals in cancer research. A limited number of anti-cancer agents directly target apoptotic pathways, and these small molecules are designed to inhibit anti-apoptotic BCL2 family members (49). Also, combining immunotherapy with agents that target the BCL2 antiapoptotic proteins may lead to a more effective treatment in melanoma (50).

In this study, ornidazole treatment significantly downregulated *Bax* expression and upregulated *Bcl2* expression compared with the control group. The increase in *Bax* expression and decrease in *Bcl2* expression was again lower in the tumor tissues of CD133^+^-injected mice than in other two groups (unsorted and CD133), which might indicate the greater resistance of CD133^+^ melanoma cells to apoptosis. On the other hand, as ornidazole treatment also downregulated the expression of Gli1, it seems reasonable to argue that ornidazole activates the apoptosis through Gli1/Bcl2/Bax-axis in melanoma cells.

We also observed significant differences in *Casp3* expression between the ornidazole-treated groups. While *Casp3* expression increased in mice that received CD133^-^ melanoma cells, it decreased in unsorted and CD133^+^ cell-injected mice compared with their control groups. Normally, increased *Casp3* activity is considered a sign of apoptosis and a positive indicator for efficient cancer treatment. However, growing evidence indicates that Casp3 promotes cancer cell growth, cellular migration, invasiveness, recurrence, and angiogenesis (51,52). We believe that this dual role of Casp3 may be related to its acting as a switch protein in important cellular pathways. However, investigating this issue was out of the scope of this study and it needs to be addressed in future studies.

The unfolded protein response (UPR) is an intracellular signaling pathway activated by the accumulation of unfolded/misfolded proteins in the ER, and thus, a vital cytoprotective mechanism in response to ER stress (53). ER stress is involved in apoptotic mechanisms leading to melanoma cell death, and ER stress-related pathways have shown to play an important role in regulating tumor formation and resistance (54). Our results demonstrated a significant increase in the expression of the cellular stress markers *Grp78* and *Xbp1*, and of ER stress-mediated apoptosis markers *Chop* and *Casp12* in ornidazole-treated groups compared with the control group. These findings suggest that to induce cellular death in melanoma cancer cells, ornidazole triggers both the Gli1/Bcl2/Bax-dependent and ER stress-mediated apoptosis pathways.

UPR signaling also activates autophagy, an evolutionarily conserved and lysosome-dependent degradation pathway in which cytoplasmic macromolecules, unfolded/misfolded proteins, damaged organelles, or pathogens are delivered to lysosomes, and are digested by lysosomal enzymes to generate ATP, nucleotides, amino acids, fatty acids, etc (55). In advanced stages of cancers, autophagy contributes to the survival and growth of the established tumors and facilitates metastasis (55). In addition, autophagy is involved in focal adhesion dynamics during cell migration and invasion. Inhibition of autophagy decreases tumor cell motility due to reduced focal adhesion turnover (56,57). In our study, ornidazole treatment significantly downregulated the expressions of ER stress-mediated autophagy markers, including *Atg5, Atg12, Becn1*, *Map1lc3b*, and *Atf4*, in all treatment groups compared with the control groups. Although these results suggest the potential of ornidazole to inhibit the autophagy mediated by ER stress in the metastatic melanoma setting, more detailed *in vivo* studies are required to analyze the influence of long-term autophagy arrest on tumor progression, metastasis, and survival.

Our findings suggest the potential of ornidazole as a novel anti-cancer agent for the treatment of malignant melanoma. Its years-long availability on the market makes ornidazole a better therapeutic candidate compared with new drugs, which require costly development. Nevertheless, larger and comprehensive studies are required to explore the safety, feasibility, and clinical effectiveness of ornidazole treatment in melanoma.

## References

[1] Kath R Rodeck U Menssen HD Mancianti ML Linnenbach AJ Elder DE Tumor progression in the human melanocytic system. Anticancer Res 1989 9 865 72 2554787

[2] Leupold D Pfeifer L Hofmann M Forschner A Wessler G Haenssle H From melanocytes to melanoma cells: characterization of the malignant transformation by four distinctly different melanin fluorescence Spectra [Review] Int J Mol Sci 2021 22 10.3390/ijms22105265 34067690 PMC8156265

[3] Riker AI Zea N Trinh T The epidemiology, prevention, and detection of melanoma. Ochsner J 2010 10 56 65 21603359 PMC3096196

[4] Ferlay J, Ervik M, Lam F, Colombet M, Mery L, Piñeros M, Znaor A, Soerjomataram I, Bray F (2020). Global cancer observatory: cancer today. Lyon, France: International Agency for Research on Cancer. Available from: https://gco.iarc.fr/today. Accessed: June 30, 2022.

[5] Domingues B Lopes JM Soares P Pópulo H Melanoma treatment in review. ImmunoTargets Ther 2018 7 35 49 10.2147/ITT.S134842 29922629 PMC5995433

[6] Tang T Eldabaje R Yang L Current status of biological therapies for the treatment of metastatic melanoma. Anticancer Res 2016 36 3229 41 27354579

[7] Parmiani G Melanoma cancer stem cells: markers and functions. Cancers (Basel) 2016 8 10.3390/cancers8030034 26978405 PMC4810118

[8] Zhang X Wang W Wang Y Jiang G Identification of genes and pathways leading to metastasis and poor prognosis in melanoma. Aging (Albany NY) 2021 13 22474 89 10.18632/aging.203554 34582363 PMC8507267

[9] Soengas MS Lowe SW Apoptosis and melanoma chemoresistance. Oncogene 2003 22 3138 51 10.1038/sj.onc.1206454 12789290

[10] Schatton T Murphy GF Frank NY Yamaura K Waaga-Gasser AM Gasser M Identification of cells initiating human melanomas. Nature 2008 451 345 9 10.1038/nature06489 18202660 PMC3660705

[11] Nandy SB Lakshmanaswamy R Cancer stem cells and metastasis. Prog Mol Biol Transl Sci 2017 151 137 76 10.1016/bs.pmbts.2017.07.007 29096892

[12] Huang T Song X Xu D Tiek D Goenka A Wu B Stem cell programs in cancer initiation, progression, and therapy resistance. Theranostics 2020 10 8721 43 10.7150/thno.41648 32754274 PMC7392012

[13] Sun S Qiu XS Cancer stem cells and tumor metastasis. J Cancer Res Ther 2013 9 Suppl S150 2 10.4103/0973-1482.122510 24516051

[14] Espinosa-Sánchez A Suárez-Martínez E Sánchez-Díaz L Carnero A Therapeutic Targeting of Signaling Pathways Related to Cancer Stemness. Front Oncol 2020 10 1533 10.3389/fonc.2020.01533 32984007 PMC7479251

[15] Shakhova O Sommer L Testing the cancer stem cell hypothesis in melanoma: the clinics will tell. Cancer Lett 2013 338 74 81 10.1016/j.canlet.2012.10.009 23073475

[16] Welte Y Davies C Schäfer R Regenbrecht CR Patient derived cell culture and isolation of CD133^+^ putative cancer stem cells from melanoma. J Vis Exp 2013 73 e50200 10.3791/50200 23525090 PMC3636786

[17] Al Dhaybi R Sartelet H Powell J Kokta V Expression of CD133+ cancer stem cells in childhood malignant melanoma and its correlation with metastasis. Mod Pathol 2010 23 376 80 10.1038/modpathol.2009.163 20062010

[18] Simbulan-Rosenthal CM Gaur A Zhou HAbdusSamad M, Qin Q, Dougherty R, et alCD133 is associated with increased melanoma cell survival after multikinase inhibition. J Oncol 2019 2019 6486173 10.1155/2019/6486173 31379943 PMC6662463

[19] Maresca L Stecca B Carrassa L Novel therapeutic approaches with DNA damage response inhibitors for melanoma treatment. Cells 2022 11 10.3390/cells11091466 35563772 PMC9099918

[20] Rappa G Mercapide J Anzanello F Le TT Johlfs MG Fiscus RR Wnt interaction and extracellular release of prominin-1/CD133 in human malignant melanoma cells. Exp Cell Res 2013 319 810 9 10.1016/j.yexcr.2013.01.003 23318676 PMC3594006

[21] Jia Y Wang Y Xie J The Hedgehog pathway: role in cell differentiation, polarity and proliferation. Arch Toxicol 2015 89 179 91 10.1007/s00204-014-1433-1 25559776 PMC4630008

[22] Gupta S Takebe N Lorusso P Targeting the Hedgehog pathway in cancer. Ther Adv Med Oncol 2010 2 237 50 10.1177/1758834010366430 21789137 PMC3126020

[23] Stecca B Mas C Clement V Zbinden M Correa R Piguet V Melanomas require HEDGEHOG-GLI signaling regulated by interactions between GLI1 and the RAS-MEK/AKT pathways. Proc Natl Acad Sci U S A 2007 104 5895 900 10.1073/pnas.0700776104 17392427 PMC1838820

[24] Bariwal J Kumar V Dong Y Mahato RI Design of Hedgehog pathway inhibitors for cancer treatment. Med Res Rev 2019 39 1137 204 10.1002/med.21555 30484872 PMC6714585

[25] O’Reilly KE de Miera EV Segura MF Friedman E Poliseno L Han SW Hedgehog pathway blockade inhibits melanoma cell growth in vitro and in vivo. Pharmaceuticals (Basel) 2013 6 1429 50 10.3390/ph6111429 24287465 PMC3854019

[26] Kubo M Nakamura M Tasaki A Yamanaka N Nakashima H Nomura M Hedgehog signaling pathway is a new therapeutic target for patients with breast cancer. Cancer Res 2004 64 6071 4 10.1158/0008-5472.CAN-04-0416 15342389

[27] Zhang J Zhang J Liu W Ge R Gao T Tian Q UBTF facilitates melanoma progression via modulating MEK1/2-ERK1/2 signalling pathways by promoting GIT1 transcription. Cancer Cell Int 2021 21 543 10.1186/s12935-021-02237-8 34663332 PMC8522148

[28] Nguyen NM Cho J Hedgehog pathway inhibitors as targeted cancer therapy and strategies to overcome drug resistance. Int J Mol Sci 2022 23 10.3390/ijms23031733 35163655 PMC8835893

[29] Ghirga F Mori M Infante P Current trends in Hedgehog signaling pathway inhibition by small molecules. Bioorg Med Chem Lett 2018 28 3131 40 10.1016/j.bmcl.2018.08.033 30177379

[30] Gonnissen A Isebaert S Haustermans K Targeting the Hedgehog signaling pathway in cancer: beyond Smoothened. Oncotarget 2015 6 13899 913 10.18632/oncotarget.4224 26053182 PMC4546439

[31] Sabbatino F Wang Y Wang X Flaherty KT Yu L Pepin D PDGFRα up-regulation mediated by sonic hedgehog pathway activation leads to BRAF inhibitor resistance in melanoma cells with BRAF mutation. Oncotarget 2014 5 1926 41 10.18632/oncotarget.1878 24732172 PMC4039118

[32] Khattak M Fisher R Turajlic S Larkin J Targeted therapy and immunotherapy in advanced melanoma: an evolving paradigm. Ther Adv Med Oncol 2013 5 105 18 10.1177/1758834012466280 23450149 PMC3556874

[33] Guo W Wang H Li C Signal pathways of melanoma and targeted therapy. Signal Transduct Target Ther 2021 6 424 10.1038/s41392-021-00827-6 34924562 PMC8685279

[34] Edwards DI Nitroimidazole drugs–action and resistance mechanisms. I. Mechanisms of action. J Antimicrob Chemother 1993 31 9 20 10.1093/jac/31.1.9 8444678

[35] Marcus Y Tal N Ronen M Carmieli R Gurevitz M The drug ornidazole inhibits photosynthesis in a different mechanism described for protozoa and anaerobic bacteria. Biochem J 2016 473 4413 26 10.1042/BCJ20160433 27647935

[36] Wang M Zhao F Li S Chang AK Jia Z Chen Y AIB1 cooperates with ERα to promote epithelial mesenchymal transition in breast cancer through SNAI1 activation. PLoS One 2013 8 e65556 10.1371/journal.pone.0065556 23762395 PMC3676316

[37] Nakamura S Umezawa H Structure of azomycin (2-nitro-imidazole). J Antibiot (Tokyo) 1955 8 66 13242482

[38] Lu Y, Liu Y, Yang C. Evaluating in vitro DNA damage using Comet assay. J Vis Exp. 2017(128). 10.3791/56450 PMC5752397 29053680

[39] Dou J Pan M Wen P Li Y Tang Q Chu L Isolation and identification of cancer stem-like cells from murine melanoma cell lines. Cell Mol Immunol 2007 4 467 72 18163959

[40] Jensen MM Jørgensen JT Binderup T Kjaer A Tumor volume in subcutaneous mouse xenografts measured by microCT is more accurate and reproducible than determined by 18F-FDG-microPET or external caliper. BMC Med Imaging 2008 8 16 10.1186/1471-2342-8-16 18925932 PMC2575188

[41] Rashid HO Yadav RK Kim HR Chae HJ ER stress: Autophagy induction, inhibition and selection. Autophagy 2015 11 1956 77 10.1080/15548627.2015.1091141 26389781 PMC4824587

[42] Pećina-Slaus N Zigmund M Kusec V Martić TN Cacić M Slaus M E-cadherin and beta-catenin expression patterns in malignant melanoma assessed by image analysis. J Cutan Pathol 2007 34 239 46 10.1111/j.1600-0560.2006.00601.x 17302608

[43] Ferretti R Bhutkar A McNamara MC Lees JA BMI1 induces an invasive signature in melanoma that promotes metastasis and chemoresistance. Genes Dev 2016 30 18 33 10.1101/gad.267757.115 26679841 PMC4701976

[44] Jeng KS Sheen IS Leu CM Tseng PH Chang CF The Role of smoothened in cancer. Int J Mol Sci 2020 21 10.3390/ijms21186863 32962123 PMC7555769

[45] Tang Y Cao Y SOX10 knockdown inhibits melanoma cell proliferation via notch signaling pathway. Cancer Manag Res 2021 13 7225 34 10.2147/CMAR.S329331 34557039 PMC8455513

[46] Girouard SD Laga AC Mihm MC Scolyer RA Thompson JF Zhan Q SOX2 contributes to melanoma cell invasion. Lab Invest 2012 92 362 70 10.1038/labinvest.2011.188 22184093 PMC3887365

[47] Santini R Pietrobono S Pandolfi S Montagnani V D’Amico M Penachioni JY SOX2 regulates self-renewal and tumorigenicity of human melanoma-initiating cells. Oncogene 2014 33 4697 708 10.1038/onc.2014.71 24681955 PMC4180644

[48] Polacheck WJ Zervantonakis IK Kamm RD Tumor cell migration in complex microenvironments. Cell Mol Life Sci 2013 70 1335 56 10.1007/s00018-012-1115-1 22926411 PMC3557537

[49] Carneiro BA El-Deiry WS Targeting apoptosis in cancer therapy. Nat Rev Clin Oncol 2020 17 395 417 10.1038/s41571-020-0341-y 32203277 PMC8211386

[50] Hersey P Zhang XD Treatment combinations targeting apoptosis to improve immunotherapy of melanoma. Cancer Immunol Immunother 2009 58 1749 59 10.1007/s00262-009-0732-5 19551381 PMC11030855

[51] Feng X Yu Y He S Cheng J Gong Y Zhang Z Dying glioma cells establish a proangiogenic microenvironment through a caspase 3 dependent mechanism. Cancer Lett 2017 385 12 20 10.1016/j.canlet.2016.10.042 27826040 PMC5323266

[52] Donato AL Huang Q Liu X Li F Zimmerman MA Li CY Caspase 3 promotes surviving melanoma tumor cell growth after cytotoxic therapy. J Invest Dermatol 2014 134 1686 92 10.1038/jid.2014.18 24434746 PMC4020991

[53] Read A Schröder M The unfolded protein response: an overview. Biology (Basel) 2021 10 33946669 10.3390/biology10050384 PMC8146082

[54] Wroblewski D Jiang CC Croft A Farrelly ML Zhang XD Hersey P OBATOCLAX and ABT-737 induce ER stress responses in human melanoma cells that limit induction of apoptosis. PLoS One 2013 8 e84073 10.1371/journal.pone.0084073 24367627 PMC3868604

[55] Li X He S Ma B Autophagy and autophagy-related proteins in cancer. Mol Cancer 2020 19 12 10.1186/s12943-020-1138-4 31969156 PMC6975070

[56] Tam SY Wu VW Law HK Influence of autophagy on the efficacy of radiotherapy. Radiat Oncol 2017 12 57 10.1186/s13014-017-0795-y 28320471 PMC5359955

[57] Lazova R Klump V Pawelek J Autophagy in cutaneous malignant melanoma. J Cutan Pathol 2010 37 256 68 10.1111/j.1600-0560.2009.01359.x 19615007

